# Residual *N*-acetyl-α-glucosaminidase activity in fibroblasts correlates with disease severity in patients with mucopolysaccharidosis type IIIB

**DOI:** 10.1007/s10545-016-9916-2

**Published:** 2016-02-23

**Authors:** O. L. M. Meijer, L. Welling, M. J. Valstar, L. H. Hoefsloot, H. T. Brüggenwirth, A. T. van der Ploeg, G. J. G. Ruijter, T. Wagemans, F. A. Wijburg, N. van Vlies

**Affiliations:** 1 Department of Pediatric Metabolic Diseases, Emma Children’s Hospital and Amsterdam Lysosome Center ‘Sphinx’, Academic Medical Center, University of Amsterdam, Meibergdreef 9, 1105 AZ Amsterdam, The Netherlands; 2 Department of Clinical Genetics, Erasmus University Medical Center, Rotterdam, The Netherlands; 3 Department of Pediatrics, Center for Lysosomal and Metabolic Diseases, Erasmus University Medical Center, Rotterdam, The Netherlands; 4 Laboratory Genetic Metabolic Diseases, Academic Medical Center, University of Amsterdam, Meibergdreef 9, 1105 AZ Amsterdam, The Netherlands; 5 Department of Pediatric Metabolic Diseases, Emma Children’s Hospital and Amsterdam Lysosome Center ‘Sphinx’ (H7-270), Academic Medical Center, University of Amsterdam, Meibergdreef 9, 1105 AZ Amsterdam, The Netherlands

## Abstract

**Background:**

Mucopolysaccharidosis type IIIB (MPS IIIB) is a rare genetic disorder in which the deficiency of the lysosomal enzyme *N*-acetyl-α-glucosaminidase (NAGLU) results in the accumulation of heparan sulfate (HS), leading to progressive neurocognitive deterioration. In MPS IIIB a wide spectrum of disease severity is seen. Due to a large allelic heterogeneity, establishing genotype-phenotype correlations is difficult. However, reliable prediction of the natural course of the disease is needed, in particular for the assessment of the efficacy of potential therapies.

**Methods:**

To identify markers that correlate with disease severity, all Dutch patients diagnosed with MPS IIIB were characterised as either rapid (RP; classical, severe phenotype) or slow progressors (SP; non-classical, less severe phenotype), based on clinical data. NAGLU activity and HS levels were measured in patients’ fibroblasts after culturing at different temperatures.

**Results:**

A small, though significant difference in NAGLU activity was measured between RP and SP patients after culturing at 37 °C (p < 0.01). Culturing at 30 °C resulted in more pronounced and significantly higher NAGLU activity levels in SP patients (p < 0.001) with a NAGLU activity of 0.58 nmol.mg-1.hr-1 calculated to be the optimal cut-off value to distinguish between the groups (sensitivity and specificity 100 %). A lower capacity of patients’ fibroblasts to increase NAGLU activity at 30 °C could significantly predict for the loss of several disease specific functions.

**Conclusion:**

NAGLU activity in fibroblasts cultured at 30 °C can be used to discriminate between RP and SP MPS IIIB patients and the capacity of cells to increase NAGLU activity at lower temperatures correlates with disease symptoms.

## Introduction

Mucopolysaccharidosis type III (MPS III or Sanfilippo disease) is a rare autosomal recessive lysosomal storage disorder in which the deficiency of one of four enzymes involved in the degradation of heparan sulfate (HS) leads to the accumulation of this specific glycosaminoglycan (GAG) (Muenzer 2011). The reported birth incidence for MPS III ranges from 1.52 to 1.89 per 100,000 newborns (Poorthuis et al 1999; Baehner et al 2005). Based on the deficient enzyme, four different MPS III subtypes are distinguished referred to as MPS IIIA (OMIM #252900), B (OMIM #252920), C (OMIM #252930) and D (OMIM #252940) (Muenzer 2011). Clinically these subtypes are indistinguishable.

The clinical course of the disease is generally divided into three stages. After a symptom-free interval, patients normally present between the age of 1 and 4 years with a delay in cognitive development, especially in the development of speech and language skills. The second phase of the disease starts at the age of 3 to 4 years and is marked by a progressive intellectual decline which is accompanied by behavioral and sleeping problems. Eventually, the third phase sets in, characterized by the regression of motor functions. Patients lose the ability to walk independently, become wheelchair bound and fully care dependent. Patients with this rapidly progressing classical phenotype (rapid progressors, RP) usually die at the end of the second or in the beginning of the third decade of life (Valstar et al 2008). However, in recent years it has been recognized that MPS III is characterized by a much broader spectrum of disease progression and severity. Survival well into adulthood has been reported and patients may show a stable developmental impairment for many years (Moog et al 2007; Valstar et al 2010a, b). In the Netherlands, most patients with MPS IIIB display this slowly progressing attenuated phenotype (slow progressors, SP) (Valstar et al 2010a).

MPS IIIB is caused by a deficiency of the enzyme *N*-acetyl-α-glucosaminidase (EC 3.2.1.50) which is responsible for the hydrolysis of the α, 1 → 4 linkage between *N*-acetylglucosamine and the adjacent glucuronic or iduronic acid residue (Esko et al 2009). The gene encoding for NAGLU is localized on chromosome 17q21.1 (Zhao et al 1996) and over 100 mutations in the NAGLU gene (HGNC: 7632) have been identified (van de Kamp et al 1976; Beesley et al 1998, 2004, 2005; Schmidtchen et al 1998; Zhao et al 1998; Bunge et al 1999; Weber et al 1999; Esposito et al 2000; Tessitore et al 2000; Emre et al 2002; Chinen et al 2005; Champion et al 2010; Valstar et al 2010a; Ouesleti et al 2011; Selmer et al 2012; Tang et al 2013). Due to this large allelic heterogeneity, establishing a genotype-phenotype correlation is difficult.

Although there is no disease modifying treatment available for MPS IIIB, several promising therapies are currently under investigation. A trial on intravenous enzyme replacement therapy (ERT) has recently been initiated (clinicaltrials.gov identifier: NCT02324049) and a study on intrathecal ERT is in a preclinical phase for MPS IIIB (http://www.bmrn.com/pipeline/index.php). The latter approach is already in a phase 2b study for MPS IIIA (clinicaltrials.gov identifiers: NCT01299727 and NCT02060526). Intracerebral gene therapy has been developed for MPS IIIB and is now in a phase 1/2 study (ISRCTN identifier: ISRCTN19853672). Furthermore, the efficacy of high dose oral genistein, which reduces the accumulation of HS in MPS III mice (Malinowska et al 2009, 2010) is being investigated in patients with all MPS III subtypes (EudraCT number: 2013-001479-18).

In order to properly target a disease modifying therapy, and to allow accurate evaluation of potential clinical benefits, it is essential to reliably predict the natural course of the disease for each individual patient at an early stage. We therefore aimed to identify markers that correlate with disease severity in MPS IIIB patients. We analyzed NAGLU activity and levels of stored HS in fibroblasts of MPS IIIB patients after culturing cells at different temperatures and correlated this to their clinical phenotype.

## Material and methods

### Patients

All Dutch patients diagnosed with MPS IIIB of whom cultured skin fibroblasts were available and essential clinical data could be retrieved from medical records, were included in this study. Data was collected on survival and the age at which the ability to speak (defined as: to verbally communicate intentionally with meaningful words) and to walk (defined as: to walk independently without any assistance) was lost. Based on the essential clinical data set, patients were divided into two groups by a clinician experienced in the diagnosis and treatment of lysosomal storage disorders, including MPS IIIB (FAW). The rapid progressor group (RP) consisted of patients with a classical, severe form of the disease and the slow progressor group (SP) of patients with a non-classical, less severe phenotype.

Informed consent for the use of patient data and fibroblasts was obtained from parents or legal representatives for all patients. This study was submitted to the Medical Ethical Review Board of the Academic Medical Center in Amsterdam who declared that ethical approval was not necessary because of the observational nature of the study.

### Mutation analysis

Mutation analysis had been performed in most patients within the scope of the diagnostic workup. If not, mutation analysis of the NAGLU gene was performed in fibroblasts by standard Sanger sequencing methods. Mutation nomenclature is according the HGVS (http://www.hgvs.org/mutnomen/). Sequence reference for the NAGLU gene is NM 000263.3.

### Cell culture

Fibroblast cell lines were tested for mycoplasma contamination and were subsequently cultured in Dulbecco’s Modified Eagle's Medium supplemented with 10 % inactivated Fetal Bovine Serum (FBS) and 100 U/mL penicillin, 100 μg/mL streptomycin, and 250 μg/mL amphotericin in a humidified atmosphere containing 5 % CO_2_. To remove bovine NAGLU in the FBS, NAGLU was inactivated by incubation of FBS at 65 °C for 35 minutes.

Fibroblasts were plated at 70 % confluence and cultured at a temperature of either 37 °C or 30 °C for 7 days. After a week the medium was removed and cell layers were washed twice with phosphate buffered saline (PBS). After harvesting, cell pellets were washed once with PBS and twice with 0.9 % NaCl and stored at −20 °C until further analysis.

### Enzymatic activity of NAGLU in fibroblasts

NAGLU activity in fibroblasts was measured using the fluorogenic substrate 4-methylumbelliferyl-2-acetamido-2-deoxy-α-D-glucopyranoside (4MU-α-GlcNAc) (Moscerdam, Oegstgeest, The Netherlands) using a modified version of the method described by Marsh and Fensom (Marsh and Fensom 1985). The 4MU-α-GlcNAc substrate was dissolved to a concentration of 6 mg/mL in 0.1 M citrate 0.2 M phosphate buffer pH 4.3. Cell pellets were resuspended in milliQ (Synergy® Water Purification System, Millipore, Billerica, MA, USA) supplemented with one cOmplete™ protease inhibitor cocktail tablet per 10 mL milliQ (Roche, Mannheim, Germany). Cells were disrupted by sonification uing a Vibra Cell sonicator (Sonics & Materials Inc., Newtown, CT, USA). Subsequently, protein concentration was measured in whole cell lysates as described by Lowry et al (Lowry et al 1951). For the determination of NAGLU activity, cell lysates were diluted to a concentration of 1 mg/mL and 30 μL of cell lysate was added to 15 μL 4MU-α-GlcNAc solution and 15 μL of a 0.1 M citrate 0.2 M phosphate buffer with pH 3.85. After 4 hours incubation at 37 °C, the reaction was stopped with 1440 μL 0.2 M sodium carbonate 0.5 M glycine buffer, pH 10.5. Released 4-methylumbelliferone was measured fluorometrically with an excitation wavelength of 360 nm and emission wavelength of 450 nm using a Perkin Elmer LS45 fluorescence spectrometer (Perkin Elmer, Waltham, MA, USA). NAGLU activity in each sample was calculated using a calibration curve of 4-methylumbelliferone (Glycosynth Ltd., Warrington, Cheshire, UK). All enzyme activity assays were performed in duplicate and repeated at least once in independent fibroblast cultures.

### HS analysis in fibroblasts

HS levels were determined as described previously (Kingma et al 2013). HS in 25 μg of fibroblast lysate was enzymatically digested into disaccharides. As a final deproteination step samples were loaded on an Amicon Ultra 10 kD centrifugal filter (EMD Millipore, Billerica, MA, USA) and centrifuged at 14,000 g for 30 minutes at a temperature of 25 °C.

### Statistical analysis

All data analyses were performed using SPSS software for Windows (version 21.0, SPSS Inc., Chicago, IL, USA). Non-parametric statistical tests were used to analyze the relationship between MPS IIIB phenotype and NAGLU activity levels after cell culturing at 37 °C and 30 °C and the NAGLU activity ratio after culturing at 30 °C over the activity after culturing at 37 °C, respectively. Linear regression was performed to determine the correlation between the ratio of NAGLU activity after cell culturing at 30 °C over 37 °C and clinical signs of disease progression. Cut-off values of NAGLU activity that could discriminate between MPS III phenotypes, were identified using receiver operating characteristic (ROC) curve analysis. True positive rates (sensitivity) were plotted against false positive rates (1-specificity) for all classification points and p-values were calculated for the area under the curve. A p-value of < 0.05 was considered statistically significant.

## Results

### Patients

Twenty-eight patients from 17 different families were included in this study (Table 1). Six patients were classified as RP patients and 22 as SP patients. At the time of this study 15 out of 28 patients were still alive. In the RP patients death occurred at a younger age than in the SP patients (median age 14 years, range 13–20 years vs. median age 51 years, range 28–69 years, respectively. p < 0.001). RP patients lost their ability to communicate in a meaningful way at a median age of 6 years (range 5–10 years). In patients with the SP phenotype this was 24 years (range 8–69 years) (p < 0.001). The median age at loss of the ability to walk independently was 14 years (range 8–18 years) in RP patients and 50 years (range 18–68 years) in the SP patients (p < 0.001).

**Table 1:** Clinical and genetic characteristics of the MPS IIIB patients. All patients for whom no data are given are still able to speak or walk. Siblings 14.2, 14.3, 14.5, and 14.6 are cousins of the siblings 14.1 and 14.4. Most patients were previously reported by Valstar et al (Valstar et al 2010a). * Patients not previously reported. ** Patients previously reported, but without mutations. ^1^ Sequence reference NM 000263.3

Patient	General information				Genetic characteristics ^1^				Clinical characteristics		
	Family	M / F	Year of birth	Phenotype	cDNA change 1.	cDNA change 2.	Protein change 1.	Protein change 2.	Age (of death)	Loss of speech	Loss of walking
Patient 1	1.1	F	1988	RP	c.214_237dup24	c.214_237dup24	p.(Ala72_Gly79dup8)	p.(Ala72_Gly79dup8)	26	5	18
Patient 2	1.2	M	1991	RP	c.214_237dup24	c.214_237dup24	p.(Ala72_Gly79dup8)	p.(Ala72_Gly79dup8)	23	8	16
Patient 3	2.1	M	1972	RP	Not identified	Not identified	Not identified	Not identified	20†	10	17
Patient 4	3.1	M	1969	RP	c.889C>T	c.217_221dup5	p.(Arg297*)	p.(Val75fs)	14†	7	11
Patient 5	3.2	F	1971	RP	c.889C>T	c.217_221dup5	p.(Arg297*)	p.(Val75fs)	13†	5	8
Patient 6	4.1	M	1963	RP	c.889C>T	c.889C>T	p.(Arg297*)	p.(Arg297*)	14†	5	9
Patient 7	5.1	M	1961	SP	c.889C>T	c.1834A>G	p.(Arg297*)	p.(Ser612Gly)	53	51	
Patient 8*	6.1	F	1979	SP	c.1834A>G	c.1927C>T	p.(Ser612Gly)	p.(Arg643Cys)	36		
Patient 9*	6.2	F	1984	SP	c.1834A>G	c.1927C>T	p.(Ser612Gly)	p.(Arg643Cys)	30		
Patient 10	7.1	F	1988	SP	c1693C>T	c.1900G>A	p.(Arg565Trp)	p.(Glu634Lys)	27	24	
Patient 11	7.2	F	1989	SP	c1693C>T	c.1900G>A	p.(Arg565Trp)	p.(Glu634Lys)	25	24	
Patient 12	8.1	M	1983	SP	c.187G>A	Not identified	p.(Asp63Asn)	Not identified	31	30	
Patient 13**	9.1	M	1988	SP	c.419A>G	c.1489C>G	p.(Tyr140Cys)	p.(Leu497Val)	26		
Patient 14	10.1	M	1997	SP	c.1834A>G	c.1834A>G	p.(Ser612Gly)	p.(Ser612Gly)	17		
Patient 15**	11.1	F	1952	SP	c.509G>A	c.743A>G	p.(Gly170Asp)	p.(His248Arg)	63	39	53
Patient 16	12.1	M	1971	SP	c.237ins24	c.1694G>A	p.(Ala72_Gly79dup8)	p.(Arg565Gln)	29†	16	19
Patient 17	12.2	M	1973	SP	c.237ins24	c.1694G>A	p.(Ala72_Gly79dup8)	p.(Arg565Gln)	42	18	18
Patient 18	13.1	F	1932	SP	c.1834A>G	c.1834A>G	p.(Ser612Gly)	p.(Ser612Gly)	69†	69	68
Patient 19	14.1	F	1947	SP	c.1927C>T	c.1927C>T	p.(Arg643Cys)	p.(Arg643Cys)	62†	24	46
Patient 20	14.2	F	1948	SP	c.1927C>T	c.1927C>T	p.(Arg643Cys)	p.(Arg643Cys)	28†	23	28
Patient 21	14.3	M	1951	SP	c.1927C>T	c.1927C>T	p.(Arg643Cys)	p.(Arg643Cys)	51†	47	49
Patient 22	14.4	F	1953	SP	c.1927C>T	c.1927C>T	p.(Arg643Cys)	p.(Arg643Cys)	58†	18	36
Patient 23	14.5	F	1954	SP	c.1927C>T	c.1927C>T	p.(Arg643Cys)	p.(Arg643Cys)	60	36	47
Patient 24	14.6	F	1956	SP	c.1927C>T	c.1927C>T	p.(Arg643Cys)	p.(Arg643Cys)	52†	19	36
Patient 25	15.1	M	1984	SP	c.845C>T	c.1172A>G	p.(Ala282Val)	p.(Tyr391Cys)	31		
Patient 26	16.1	F	1937	SP	c.1927C>T	c.281G>C + c.283G>C	p.(Arg643Cys)	p.(Arg94_Asp95delins2)	47†	8	34
Patient 27	16.2	M	1942	SP	c.1927C>T	c.281G>C + c.283G>C	p.(Arg643Cys)	p.(Arg94_Asp95delins2)	50†	8	18
Patient 28	17.1	M	1955	SP	c.1562C>T	c.1489C>G	p.(Pro521Leu)	p.(Leu497Val)	60	45	50

### Mutations

For all patients mutations are presented in Table 1. All mutations in our cohort have been reported previously, except the missense mutation c.509G > A; p.(Gly170Asp) found in patient 15 (Zhao et al 1996; Beesley et al 1998; Schmidtchen et al 1998; Weber et al 1999; Valstar et al 2010a).

### NAGLU activity in fibroblasts after culturing at 37 °C and 30 °C

**Fig. 1:**
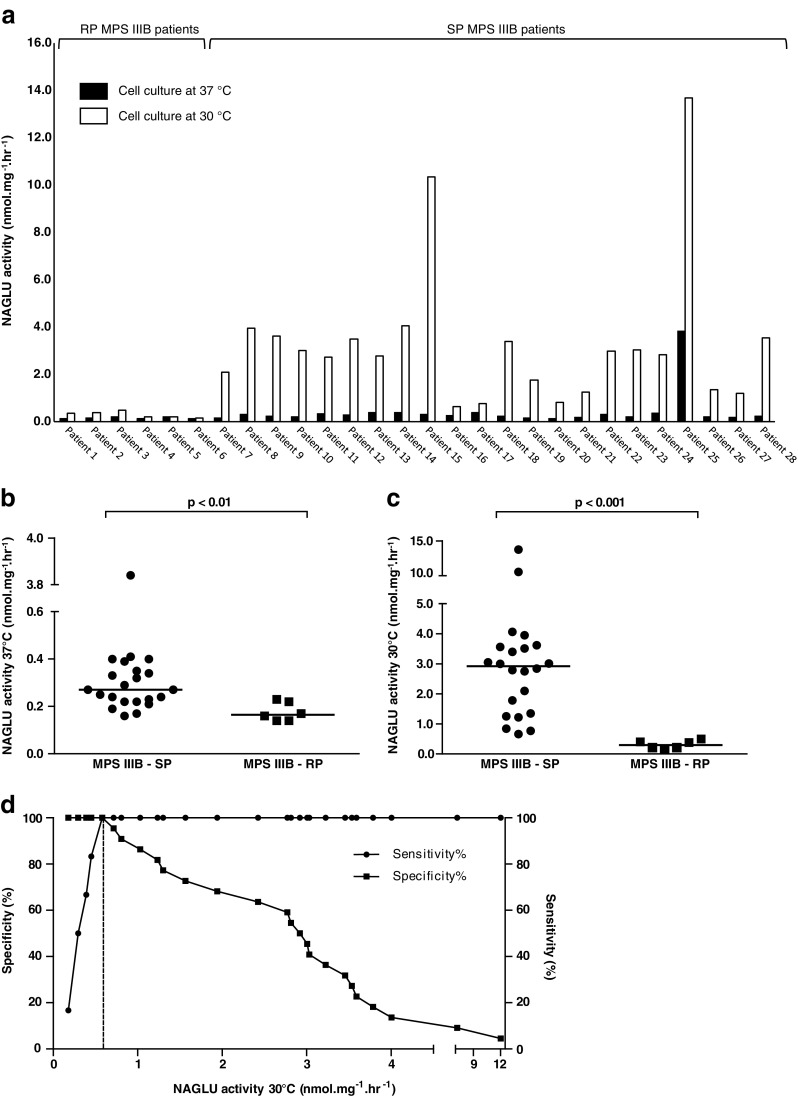
**a**. Mean NAGLU activity (nmol.mg^−1^.hr^−1^) of duplicate measurement in fibroblasts of the individual MPS IIIB patients after culturing cells at 37 °C and 30 °C for 1 week. Patient numbers correspond with the numbers in Table 1. Data of one representative experiment are shown. **b** and **c**. NAGLU activity (nmol.mg^−1^.hr^−1^) measured in fibroblasts of SP and RP MPS IIIB patients after culturing cells at 37 °C and 30 °C for 1 week, respectively. Medians are given. Data of one representative experiment are shown. **d**. Sensitivity and specificity for cut-off levels of NAGLU activity in fibroblasts after culturing at 30 °C to distinguish between RP and SP MPS IIIB patients. The dashed line indicates the calculated optimal cut-off level of 0.58 nmol.mg^−1^.hr^−1^

To investigate whether residual enzyme activity in cultured skin fibroblasts differentiates between RP and SP patients, NAGLU activity in fibroblasts was measured. In Fig. 1a, NAGLU activity levels in fibroblasts of all individual MPS IIIB patients are depicted after culturing at 37 °C and 30 °C. Very low levels of NAGLU activity were observed for all patients after culturing at 37 °C. Under these conditions fibroblasts from RP patients showed a median enzymatic activity of 0.17 nmol.mg^−1^.hr^−1^ (range 0.14-0.23 nmol.mg^−1^.hr^−1^) compared to a median enzymatic activity of 0.27 nmol.mg^−1^.hr^−1^ (range 0.16-3.84 nmol.mg^−1^.hr^−1^) in fibroblasts from SP patients. As is shown in Fig. 1b, a small, though significant difference in NAGLU activity between the RP and SP group was seen after culturing at 37 °C (p < 0.01). However, when looking at individual values of enzymatic activity there is still considerable overlap between the two groups. Figure 1c shows that culturing fibroblasts at 30 °C for 1 week resulted in a more pronounced difference in NAGLU activity between the two groups. After culturing fibroblasts at 30 °C significantly higher NAGLU activity levels were found in SP patients (median NAGLU activity 2.92 nmol.mg^−1^.hr^−1^ (range 0.66-13.70 nmol.mg^−1^.hr^−1^)) compared to RP patients (median NAGLU activity after culturing at 30 °C 0.30 nmol.mg^−1^.hr^−1^ (range 0.15-0.50 nmol.mg^−1^.hr^−1^)) (p < 0.001). ROC analysis showed an area under the curve of 1.0, indicating that NAGLU activity after culturing at 30 °C is a very accurate tool to discriminate between RP and SP patients (p < 0.001). A NAGLU activity of 0.58 nmol.mg^−1^.hr^−1^ was calculated to be the optimal cut-off value to distinguish RP patients from SP patients with a sensitivity and specificity of 100 % (Fig. 1d).

### HS levels in fibroblasts

**Fig. 2:**
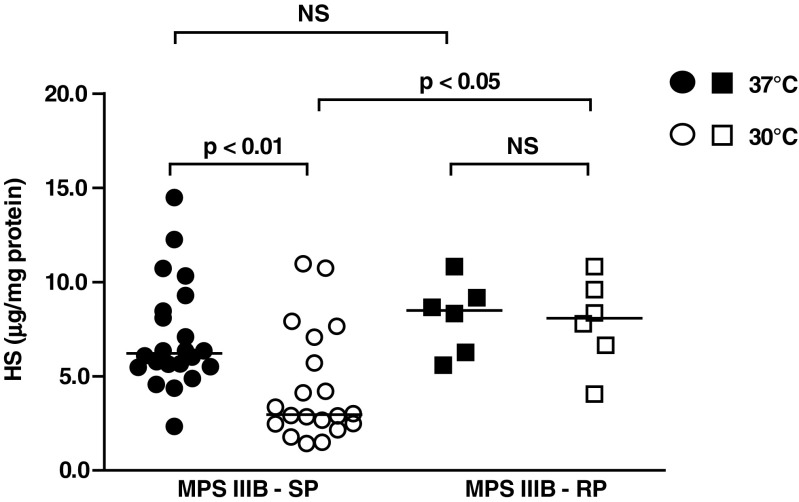
HS levels (μg/mg protein) in fibroblasts of SP and RP MPS IIIB patients after culturing cells at 37 °C and 30 °C. Medians are given. Data of one representative experiment are shown

HS levels in RP and SP MPS IIIB patients’ fibroblasts were analyzed to assess whether storage levels are influenced by the differences in NAGLU activity levels observed between the two groups. No significant difference was found in HS level between the groups when fibroblasts were cultured at 37 °C (Fig. 2). However, in fibroblasts cultured at 30 °C HS levels were significantly lower in fibroblasts from patients with an SP phenotype compared to fibroblasts from patients with an RP phenotype (p < 0.05). Culturing fibroblasts of RP patients at either 37 °C or 30 °C, did not result in any significant differences in HS levels, while significantly lower HS levels after culturing at 30 °C were found in patients within the SP group, compared to levels after culturing at 37 °C (p < 0.01). This indicates that the increased NAGLU activity, measured *in vitro* after culturing cells at 30 °C, indeed corresponds to higher levels of functional NAGLU in fibroblasts of SP patients.

### Disease symptoms and NAGLU activity ratio after culturing at 30 °C over 37 °C

**Fig. 3:**
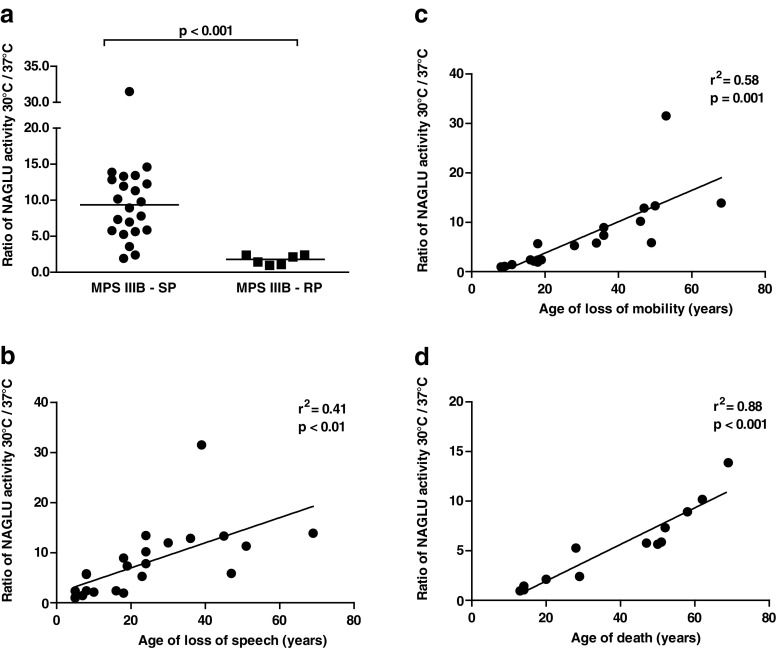
**a**. Ratio of NAGLU activity after culturing at 30 °C over the activity after culturing at 37 °C measured in fibroblasts of SP and RP MPS IIIB patients. Medians are given. Data of one representative experiment are shown. **b**-**d**. Ratio of NAGLU activity after culturing at 30 °C over the activity after culturing at 37 °C in fibroblasts, correlated to the age of speech loss (23 out of 28 patients), to the age of loss of mobility (19 out of 28 patients), and correlated to the age of demise (13 out of 28 patients who had died at time of this study). Data of one representative experiment are shown

The capacity of patients’ fibroblasts to increase residual enzyme activity was further assessed by calculating the ratio of NAGLU activity after culturing at 30 °C over the activity after culturing at 37 °C. The ratio of NAGLU activity after culturing at 30 °C over 37 °C was 9.36 (range 1.93-31.49) in SP patients, which was significantly higher compared to patients in the RP group, who had a median ratio of NAGLU activity of 1.78 (range 0.96-2.39) (p < 0.001) (Fig. 3a). Because MPS IIIB comprises a continuous spectrum of disease severity, we assessed whether this ratio correlated more specifically with the age at which patients lost specific functions. A lower capacity of patients’ fibroblasts to increase NAGLU activity at 30 °C could significantly predict for the loss of the ability to communicate verbally in a meaningful manner and the loss of the ability to walk independently at a younger age (Fig. 3b and c). Also, the age of demise correlated with the NAGLU activity ratio after culturing at 30 °C over the activity after culturing at 37 °C (Fig. 3d).

## Discussion

Over recent years, a number of studies on potential disease modifying treatment options for MPS IIIB have been initiated. For evaluating clinical efficacy as well as for assessing which patients may benefit the most from a specific treatment, it will be essential to predict the natural course of the disease for each individual patient at an early stage. Here, we investigate an approach to discriminate between MPS IIIB patients with a rapidly progressing (RP, classical or severe) phenotype and patients with a slowly progressing (SP, attenuated) phenotype, using cultured skin fibroblasts.

We observed a significant difference in residual activity of NAGLU between fibroblasts from RP and SP patients when fibroblasts were cultured at 37 °C. However, NAGLU activity did not completely discriminate between the two groups. Culturing fibroblasts at 30 °C, however, allowed for complete separation between RP and SP patients. Accordingly, HS levels after culturing at 30 °C were significantly lower in fibroblasts from the SP group than in fibroblasts from the RP group. This indicates that the increased NAGLU activity measured in protein lysates after culture at 30 °C comprises a form of the enzyme that is biochemically active in living cells and exerts its catalytic function in the lysosome. Nevertheless, it should be taken into consideration that other factors, such as alteration of GAG synthesis, are responsible for lower HS levels. However, it is unlikely that this only affects the SP group.

MPS IIIB is characterized by a large genetic heterogeneity which is probably the most important cause of the phenotypic variability. Although some genotype-phenotype correlations have been established in MPS IIIB, previously unrecognized mutations are frequently reported. In our limited series of patients, the RP patients all have two nonsense or frameshift mutations, whereas all the SP patients have at least one missense mutation. Irrespectively of genotype, NAGLU activity in fibroblasts after culturing at 30 °C appears to reliably predict for an RP or SP phenotype.

MPS IIIB comprises a continuous spectrum of disease severity, and a division in only an RP and an SP group does no justice to the clinical variability observed in patients. Valstar et al showed that the loss to communicate in a meaningful manner and the ability to walk independently are key symptoms in the assessment of disease progression (Valstar et al 2010a). We therefore correlated the loss of these functions to the ratio of NAGLU activity at 30 °C over 37 °C. The capacity of fibroblasts to enhance residual enzyme activity at 30 °C correlated with the course of the disease. However, since not all patients had already reached these stages of disease, patient numbers are small and more patients would be needed to validate these correlations.

The increased levels of residual NAGLU measured in SP patients after culturing cells at 30 °C, might be due to more efficient protein folding at this lower temperature, thereby stimulating the activity of mutant enzymes (Gootjes et al 2004; Diekman et al 2015). The positive effect of culturing human fibroblasts at 30 °C on protein quantity and enzymatic activity has already been demonstrated for a distinct metabolic disorder by another group in our lab (Houten et al 2002). Cells of patients with a mild mevalonate kinase deficiency showed higher enzymatic activity after culturing at 30 °C which correlated with higher protein levels on Western blot. Culture temperature had no effect on cells of patients with a severe mevalonate kinase deficiency. We hypothesize that the higher levels of NAGLU activity found in SP patients might be caused by the presence of molecular chaperones or differences in the regulation of the endoplasmic-reticulum-associated protein degradation machinery which only become apparent at 30 °C culture conditions, at least in the *in vitro* setting.

In most SP fibroblasts, culturing at 30 °C resulted in levels of NAGLU activity that exceeded 10 % of control enzyme activity (reference values for NAGLU activity in fibroblasts used in our diagnostic laboratory are 9–17 nmol.mg^−1^.hr^−1^). Ten percent of control NAGLU activity is often associated with the amelioration of symptoms. Our findings indicate that residual NAGLU activity can potentially be enhanced and that MPS IIIB patients with a SP phenotype might benefit from therapies that interfere with protein folding, such as chemical and pharmacological chaperones (Meijer et al 2013). However, more research is needed to elucidate the underlying processes and the effects of therapies that interfere with these mechanisms.

To be able to measure low levels of NAGLU activity and to detect minor changes in enzyme activity at different conditions in all patient cell lines, we optimized the assay normally used in our diagnostic department. Using this optimized assay, in patient 25 remarkably high levels of NAGLU activity and low HS levels were found at all culture conditions, despite previous genetic and biochemical confirmation of the diagnosis MPS IIIB. The use of high concentrations of 4MU-α-G1cNAc substrate (1.5 mg/mL final concentration) in combination with an optimized pH and incubation time, might have favored the particular kinetic properties of the mutant enzyme in this patient. Furthermore, NAGLU with this particular mutation might have an increased affinity to the 4MU-substrate, as compared to natural substrates present *in vivo*. This is currently being further investigated.

In conclusion, we show that NAGLU activity in fibroblasts cultured at 30 °C can be used to discriminate between RP and SP MPS IIIB patients and that the capacity of cells to increase NAGLU activity at lower temperatures correlates with disease severity and progression. Prediction of the phenotype of an individual patient may become of high relevance in the near future for assessment of the efficacy of disease modifying treatments for MPS IIIB.
